# A historical and ethnobotanical study on local botanical knowledge recorded in the book “*Mongolia and Amdo and the Dead City of Khara-Khoto*”

**DOI:** 10.1186/s13002-021-00443-2

**Published:** 2021-05-25

**Authors:** Guixi Liu, Wurheng, Yanying Zhang, Shirong Guo, Yongmei, Khasbagan

**Affiliations:** 1 Institute for the History of Science and Technology, Inner Mongolia Normal University, Hohhot, 010022 China; 2 College of Life Science and Technology, Inner Mongolia Normal University, Hohhot, 010022 China; 3 Key Laboratory Breeding Base for Biodiversity Conservation and Sustainable Use of Colleges and Universities in Inner Mongolia Autonomous Region, Hohhot, China

**Keywords:** P. K. Kozlov, Expedition record, Local knowledge on plants, Mongolian folk, Ethnobotany, Botanical history

## Abstract

**Background:**

There is a plentiful amount of local knowledge on plants hidden in the literature of foreign exploration to China in modern history. *Mongolia and Amdo and the Dead City of Khara-Khoto* (MAKK) is an expedition record on the sixth scientific expedition to northwestern China (1907–1909) initiated by P. K. Kozlov (1863–1935), a famous Russian Central Asian explorer. Used as a non-professional biology book, MAKK contains some botanical knowledge. The information noted down over more than 100 years ago is about the traditional knowledge of the Mongolian folks lived on the Mongolian plateau and the Qinghai-Tibet Plateau for the understanding and utilization of plants, which is of a highlighted function for the study of the botany and the history of science and technology. We therefore have carried on relevant collation, analysis, investigation and criticism to Mongolian local knowledge on plants in MAKK, and obtained the status quo of these local knowledge.

**Methods:**

The authors used the literature research method to sort and compare the two versions of MAKK, separating out the Mongolian local knowledge about plant naming and utilization. Then, these contents were verified through literature textual method and were catalogued according to the method of ethnobotany. Based on these, the authors carried out field investigations along with Kozlov’s expedition routes in Alxa in 2019 and 2020, respectively. The methods of key informants interview, snowball sampling, and rational sampling were all used in field investigations. By analyzing the interview data of 34 key informants, we obtained the status quo of local knowledge recorded in MAKK.

**Results:**

By means of regulation and research, it is found that Mongolian plant folk names of one genus and eight species were recorded in MAKK. Their morphological characteristics and traditional grazing knowledge are crucial naming basis. There are three types on the structures of Mongolian plant name: simple primary name, complex primary name, and secondary name. Corresponding relations between Mongolian folk name and scientific name are existed in “one-to-one,” “multitude-to-one,” and “one-to-multitude” forms. The classification of certain plants by Mongolian people has reached the level of species or varieties.

In addition, the Mongols’ usage for nine species of plants was noted in MAKK. These plants are mainly used for edible, graziery, fuelwood, building material, toponym, and belief. With the development and change of the society, it is found that some utilization methods have been replaced or even disappeared, while the remainders still continue to be applied.

**Conclusions:**

Firstly, the Mongols have indigenous rules and systems for nominating and classifying plants. Secondly, the Mongolian local knowledge on plants possesses multiform character. Thirdly, the Mongolian local knowledge on plants and Mongolian culture have mutual influence and interdependence relationship. Fourthly, the Mongolian local knowledge on plants urgently needs to be protected in many forms. Finally, it is veritable and reliable for the records of Mongolian botanical local knowledge in MAKK by textual research, and it is valuable for scientific research. The historical notes more than 100 years ago are not only supply dependable information and momentous historical data for Mongolian ethnobotany and Chinese minority science and technology history research, but also offer references for ecology, flora, and botanical history study.

## Background

As typical nomadic people, Mongolians have mainly live on the Mongolian Plateau, Qinghai-Tibet Plateau, and other large areas of Central Asia. Mongolians gradually form unique local knowledge on botany during the long-term exploration and understanding of the natural environment and resources. A summary of most knowledge obtained from practical experience can be attributed to the comprehension of natural matters and the laws of nature. From the perspective of modern science, it belongs to the research category of ethnobotany. The local knowledge for plants is one of the traditional knowledge of Mongolians, and it concerns the naming and usages of plants perceived by the local or indigenous people existing in a given area [1]. Apart from passing on from generation to generation in the folk by words or by doing, the knowledge is also noted down in many documents and books [2, 3]. The literature chiefly includes historical records, medical books, chorography, travel notes, and so on. Although these books and documents are manifold, they showcase traditional knowledge and experience which are of extreme significance for the study of ethnobotany, plant diversity and the history of science and technology [4–6].

Since modern times, foreigners have carried out frequent inspections in China and recorded a great deal of Mongolian folk traditional knowledge in their investigation works. Discouragingly, the literature has not attracted sufficient attention from ethnobotany researchers to date. With the evolution and changes of the community, the lifestyle of Mongolians undergoes the variety from nomadism to settlement. Simultaneously, a large amount of traditional knowledge is being slipped away at an alarming rate, which is relevant to botany due to lack of inherited condition and need [1, 7, 8]. It is thus thoroughly impending and indispensable for regulating and getting a deep insight into the literature of foreigners’ visit to China in modern times.

**Fig. 1:**
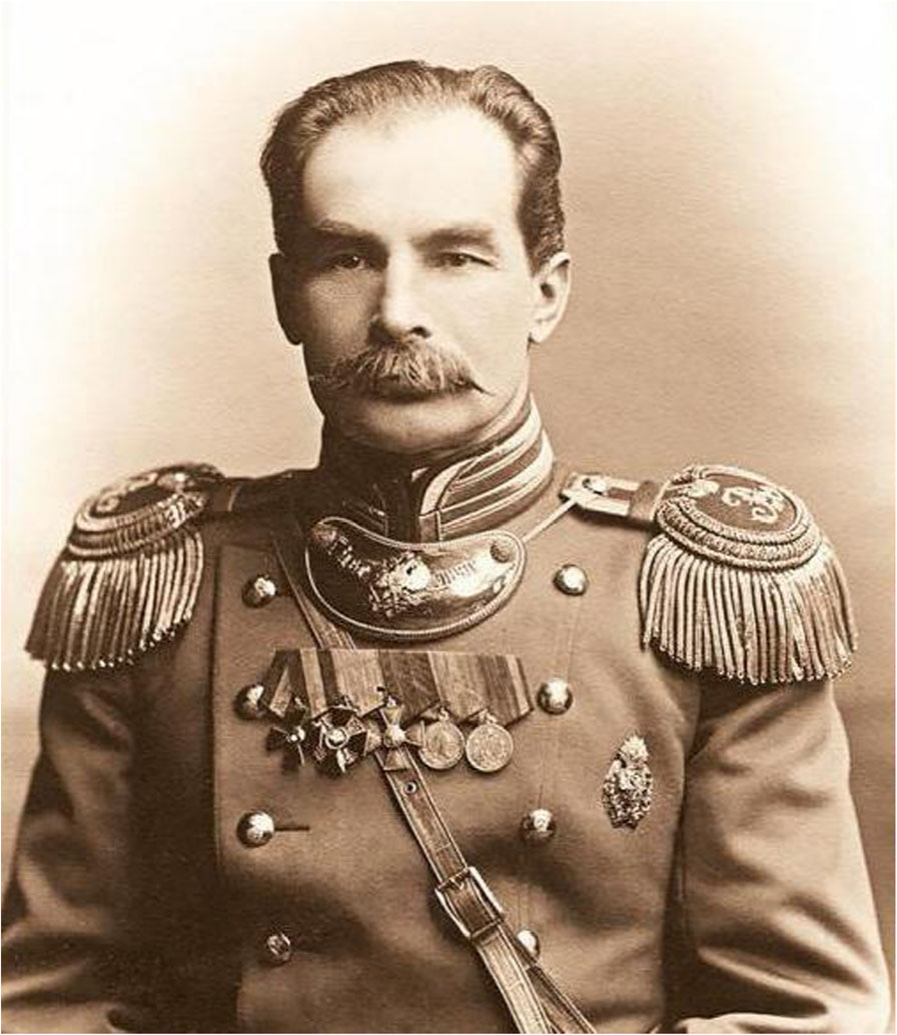
P. K. Kozlov (1863-1935). (https://fb.ru/misc/i/gallery/27428/1728454.jpg)

Pyotr Kuz’mich Kozlov (Петр Кузьмич Козлов, 1863–1935) was a Russian explorer, archaeologist, and a renowned central Asiatic comprehensive explorer [9–14] (Fig. 1). He was known for the discovery of Harahot, the site of the Tangut Era Black Water City in Ejina Banner, Inner Mongolia, China [15]. He owned seven chances to execute scientific expeditions to China and his sixth travel was conducted from 1907 to 1909 [16]. Apart from the investigation to explore Harahot ancient city, the team also carried out an all-sided and painstaking research job. Adopting the field investigation method, the team inspected and recorded in depth for the natural ecological environment, ethnic social culture as well as traditional knowledge of Mongolian botany in Mongolia area (now called as Mongolia and Inner Mongolia of China) and Qinghai Region. All the contents were primarily contained in his expedition note—*Mongolia and Amdo and the Dead City of Khara-Khoto* (MAKK, 《Монголия и Амдо и мертвый город Хара-хото》).

**Fig. 2:**
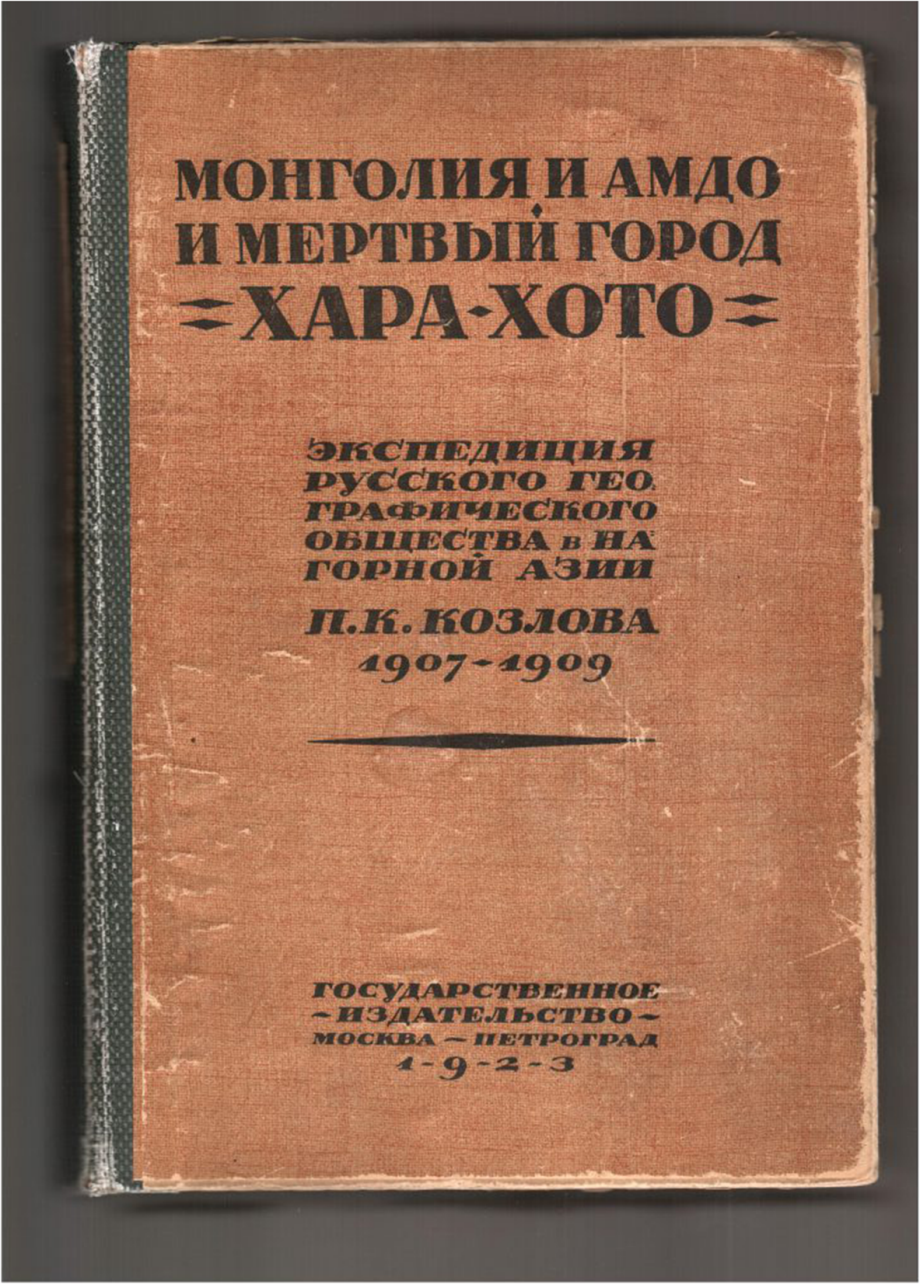
Original edition of MAKK (1923). (http://kozlov-museum.ru/wp-content/uploads/2016/07/3-218x300.jpg)

**Fig. 3:**
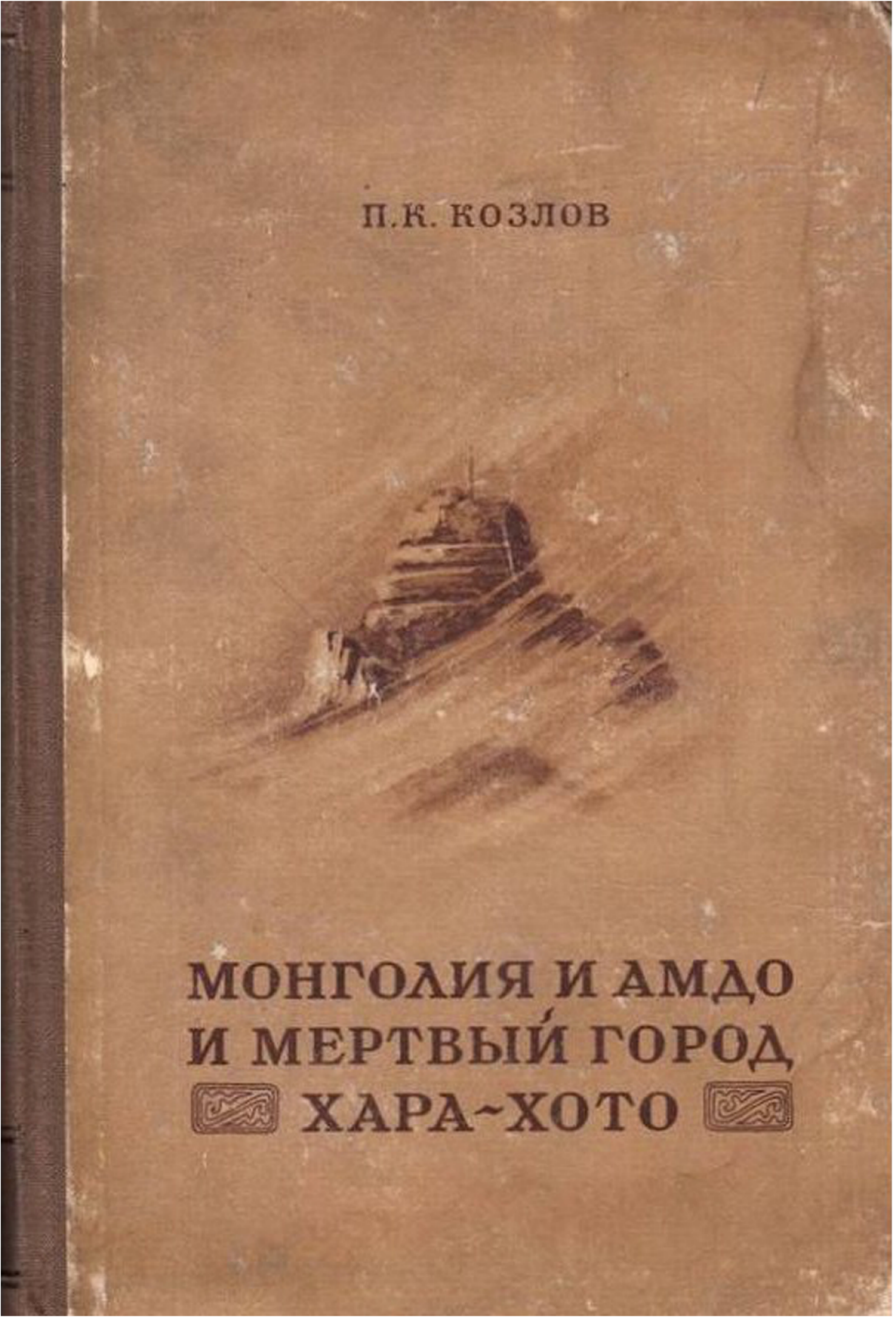
Second edition of MAKK (1948). (https://www.svetanaknigite.com/51011-hickbox_default/mongoliya-i-amdo-i-mertviy-gorod-hara-hoto-1948-g.jpg)

MAKK was published by State Geographical Literature Publishing House of the Soviet Union in 1923 [17] (Fig. 2) and republished in 1948 [18] (Fig. 3). To date, the work has been translated into English, Italian, German, Mongolian, Chinese, and many other languages [19, 20]. There are three editions of Chinese translation: *Journey to the Dead City* (Guixing Chen, 2001) [21], *Mongolia, Amdo and The Dead city of Harahot* (Xilong Wang, Shuqing Ding, 2002) [22], and *Mongolia, Amdo and The Dead city of Harahot (full version)* (Xilong Wang, Shuqing Ding, 2011) [23].

**Fig. 4:**
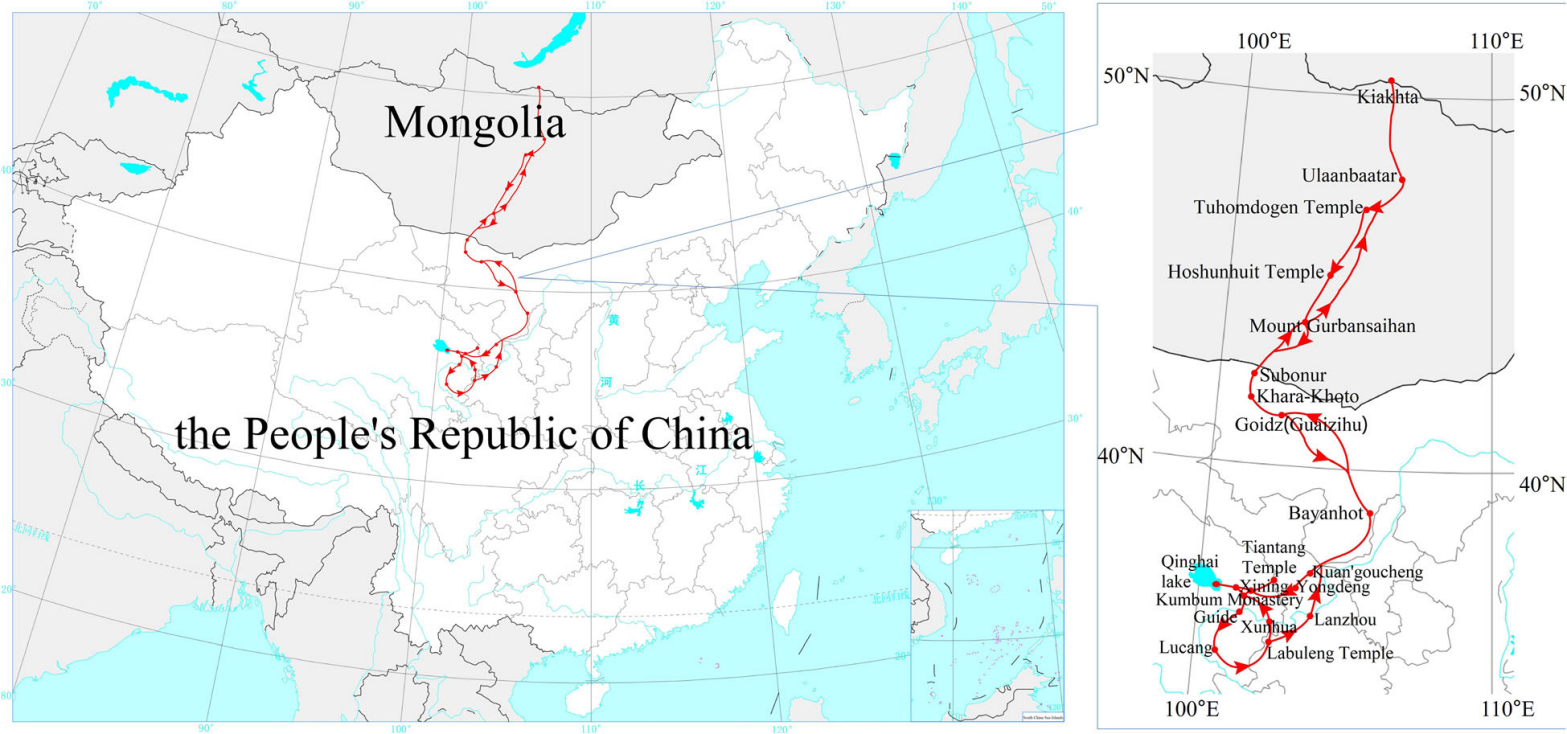
Kozlov’s expedition routes. (Drawn by Muyi Rou and Guixi Liu)

The Kozlov’s expedition region covered the central and southern Mongolian plateau, the northeast Qinghai-Tibet Plateau, which belongs to the desert and plateau areas in the interior of Asia. Noteworthily, their geographical and ecological environment is complicated and diversiform. The inspection area has a large span and the range is approximately 100°E~107°E, 34.7°N~50.3°N (Fig. 4).

The routes of the Kozlov’s expedition could be divided into three sections: (a) The expedition team had headed south from Kyakhta to Gulban-saihan mountain of the Gobi Altai Mountains, passing Kulun (Ulaanbaatar now). (b) The team members had crossed over Gulban-saihan Mountain into Ejina Banner of Inner Mongolia. Then they had followed the Ejina River upstream from Subonur (East Juyan Lake Basin now) to the ancient city of Harahot (the Black City Ruins now).

Soon after going to the east, passing through Goidz (Wentugaole now) and walking along the northeast edge of Badain Jaran Desert, they finally reached Dingyuanying (Bayanhot now), and continued to investigate Ho-lan Mountains. Kozlov’s team had left Inner Mongolia along the southeast edge of Tengger Desert 2 months later. (c) The main party of the expedition had passed through Pingfan (Yongdeng County now), traveled across the Qilian Mountains, gone up the Huangshui River, and arrived at Xining. In the meanwhile, they had been inspecting Kumbum Monastery (Ta’er Lamasery now), Qinghai Lake, Guide as well as Amdo Tibetan areas on the way. In the end, Kozlov’s expedition had basically went back over the same route.

The Kozlov’s team crossed the Gobi Desert in the central Mongolian plateau and reached the northern Tibetan side of Qinghai-Tibet Plateau, passing through the districts belonged to Khalkha Mongol and Olot Mongol. Specifically, it mainly refers to the Tushiyetu Khan aimag and Sayinoyan tribe of Khalkha Mongolia, the Ejina Torgut tribe of the Olot Mongolia [24], Alxa Khoshud Deparment [25, 26], together with the Khoshud Mongol regions in Gansu and Qinghai provinces [27–33]. There are a crowd of ethnic groups in the Kozlov’s investigation region and the traditional Mongolian culture is more or less affected and infiltrated by other ethnic cultures (especially the farming culture of the Han nationality). Nevertheless, Mongolians lived in these regions had not been greatly influenced by the development of modern civilization in the early twentieth century. They had been sticking to traditional nomadic lifestyles during quite a long period, bringing about the relatively integrated preservation and inheritance of traditional knowledge and culture in folk.

On account of a number of branches widely distributed, it is somewhat different that the folk culture and customs of the Mongolian people in diverse areas or branches. Additionally, the distribution of plants derived from disparate geographical environment is not the same, causing their comprehension and usage of plants some peculiarity. All in all, the culture customs along with the cognition and utilization of plants among the same nationality have more in common and own a mass of consistency. On the basis of Xiaotong Fei’s theory of “multiple integration” [34], Shengji Pei et al. [35] figured out that there is diversity and unity on the aspect of making use of conventional knowledge on plants. The work of Soyolt and Khasbagan et al. [36] furtherly testified botanical local knowledge of the Mongols possesses regional and unified characteristic. The study therefore can reflect the interrelation between Mongolians and plants in a specific region to some extent.

Since Kozlov was not a botanist; his expedition was not a specialized ethnobiology survey and MAKK is not a special work of botany. Hence, the academic circle in botany paid little attention to MAKK. Although there is some information about Mongolian understanding and utilization of plants in MAKK, it is only a small part of botanical knowledge of the Mongols at that time. As it was collected occasionally along with the investigation and was very scattered, so it is difficult to sort out and study. This may be one of the reasons why no ethnobotany research has been conducted on them up to now. Nevertheless, the botanical information of more than 100 years ago noted in MAKK has crucial academic value. It provides fundamental historical data for the current Mongolian ethnobotany research, plant diversity research, and Chinese ethnic minorities’ science and technology history research. Whether the local knowledge still exists to date or not requires us to conduct targeted investigation. Hence, it is imperative to comb and analyze the contents of Mongolian folk botany in MAKK.

## Materials and methods

### Materials

#### Selection of material

We have been very interested in the historical records of Mongolian understanding and utilization of local plants. In particular, the records of foreign investigators provide valuable information supplements. The Russians made relatively rich investigations and kept detailed records of the areas inhabited by the Mongols among the investigations to China in modern times. Since Kozlov came to China more times among Russian explorers, and there are many records of Mongolian local knowledge of plants in his survey notes, we first choose his famous expedition records (MAKK) to attempt to study.

In this article, we take the two Russian editions (1923 and 1948) of MAKK as the chief study materials, select the Mongolian local knowledge about plants noted as the research object, and then conduct collation, textual research and investigation.

#### Field study area

**Fig. 5:**
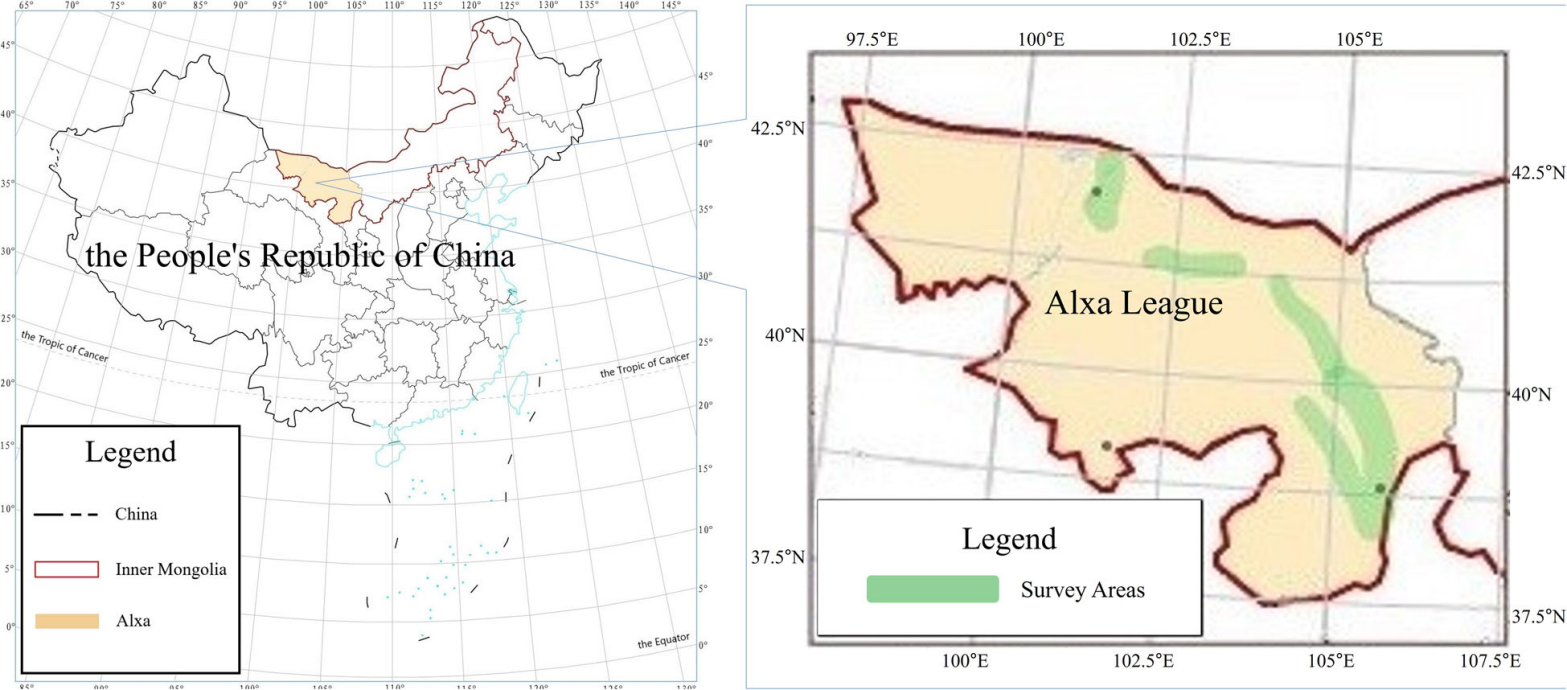
Field survey areas. (Drawn by Muyi Rou and Guixi Liu)

As the geographical span of Kozlov’s expedition is excessively vast, it is not possible to survey all the locations recorded. Fortunately, the local knowledge of Mongolian botany mentioned in MAKK is primarily concentrated in Alxa, Inner Mongolia and its adjacent regions. Hence, the paper selects Alxa League in Inner Mongolia Autonomous Region of China as the main district for field investigation based on the objective conditions. The field survey areas are shown in Fig. 5.

### Methods

The main research methods of the article are literature research, textual research, field investigation, and semi-structured interview.

- The relevant information on botanical local knowledge of Mongolian should be picked out by combing and comparing with two Russian versions of MAKK.
- *Flora of China* [37–46], *Flora of Inner Mongolia (third edition)* [47], *Mongolian-Chinese Series of Terms of Natural Science Botany* [48], Mongolian*-Russian-Latin-Chinese Name of Plants* [49], *Mongolian-Russian-Latin-Chinese Checklist of Mongolia Flora* [50], besides some main reference books [51–55] and relative literature [56–62], along with the scientific databases such as Species 2000, ITIS and Iplant, the message over the naming and utilization of plants organized were verified and cataloged.
- During the Kozlov’s investigation month, we carried out along the routes of his inspection in Alxa by field investigations in 2019 and 2020, respectively. According to the records in MAKK, the interviews were conducted with the local Mongolians to further verify the accuracy of Kozlov’s records and investigate the status quo of those knowledge. A total of 34 key informants participated in interviews via selection using snowball sampling and rational sampling [63, 64]. Since eight of them are Mongolians living in Ordos, Inner Mongolia, as well as Qinghai and Xinjiang, we conducted the interviews by telephone. The key informants are herders, scholars, civil servants, teachers, vendors, and students, and the ages of them ranged from 26 to 76.

## Results and discussion

By organizing information of plants in MAKK, it was found that Kozlov set down not only the Mongolian and Latin scientific names of some plants, but their habitats, morphological characteristics, usages and utilization methods, and so on. Based on analysis and textual research, MAKK recorded the Mongolian folk names for 1 genus and 8 species of plants (Table 1), and the usage of 10 species by Mongols (Table 2). The botanical local knowledge involves 14 species of plants in all, belonging to 14 genera of 11 families.

**Table 1:** Plants named in the Mongolian language recorded in MAKK

Original record name	Corresponding scientific acceptance name	Corresponding folk name	Meaning	Morphological characteristics
Тограк (*Populus euphratica*)	*Populus euphratica* Oliv.	[towray]/tɔ:rɔi/	PN	–
Хайлис (-)	*Ulmus pumila* L.	[xayilasu]/xɑils/	PN	–
Сульхир (*Agriophyllum gobicum*)	*Agriophyllum squarrosum* (L.) Moq.	[sulhir]/sʊlhir/	PN	–
Хату-хара (-)	*Amygdalus mongolica* (Maxim.) Ricker	[xatagu xar_a]/xɑtʊ: xɑr/	Hard black	Branches: hard wood, gray-black in color
		^*^ [ulagan buyilasu]/ʊlɑːn-bʊɪls/	Red *Amygdalus pedunculata*	Flower: red
		^*^ [xar_a modo]/xɑr mɔd/	Black tree	Branches: gray-black in color
		^*^ [xar_a buta]/xɑr bʊt/	Black bush	Branches: gray-black in color
Карагана (*Caragana*)	*Caragana* Fabr.	[xargan_a]/xɑrgɑnɑː/	PN	–
Хармык (*Nitraria schoberi*)	*Nitraria tangutorum* Bobr.	[xarmag]/xɑrmɑg/	PN	–
		^*^ [bögereg]/bo:rog/	PN	–
		^*^ [usun üsüg]/ʊsn usəg/	Water-rich Sour	Berries, juicy Taste:sour
Дзрзсун (*Lasiagrostis splendens*)	*Achnatherum splendens* (Trin.) Nevski	[deresü]/dərs/	PN	–
Мото-ширик (*Kobresia thibetica*)	*Kobresia tibetica* Maxim.	[modo sirigi]/mɔd ʃirəg/	Wood grass	Culms: rigid and erect, like wood
Цакэлдак (-)	*Iris lactea* var. *chinensis* (Fisch.) Koidz.	[čaxildag]/tʃæxʲɑldɑg/	PN	–
		^*^ [čaxirm_a]/tʃæxʲɑrmɑː/	PN	–

^*^ The folk Mongolian name obtained by interview.

*PN* primary name, no other meaning.

The species in the inventory are arranged according to the Engler system.

The Mongolian folk names are spelled in Uygur Mongolian and marked with international phonetic symbols [65].The phonetic symbols of written language are in square brackets, and the spoken language ones are in the double slash.

**Table 2:** Usage information of plants among Mongolian folk recorded in MAKK

Usage	Scientific name	Parts	Methods
**Edible**	*Agriophyllum squarrosum* (L.) Moq.	Seed	Substitute of grain, grinding into powder, Steamed or Fried
	*Potentilla anserine* L.	Root tuber	–
**Graziery**	*Haloxylon ammodendron* (C. A. Mey.) Bunge	Browse	Forage, Feeded camel
	*Achnatherum inebrians* (Hance) Keng	Whole plant	Poisonous plant, Prevent poisoning of horses after ingestion
**Fuelwood**	*Haloxylon ammodendron* (C. A. Mey.) Bunge	Whole plant	Fuel, burned
**Building material**	*Phragmites australis* (Cav.) Trin. ex Steud.	Haulm and leaf	Mixed with mud to build wall or made into bricks
	*Haloxylon ammodendron* (C. A. Mey.) Bunge	Trunk and branch, or whole plant	Built sheds for livestock, pile into wall or Obo
	*Populus euphratica* Oliv.	Trunk and branch, or whole plant	Built sheds for livestock, pile into wall or Obo
	*Ulmus pumila* L.	Trunk and branch, or whole plant	Built sheds for livestock, pile into wall or Obo
**Toponym**	*Ulmus pumila* L.	–	Named as place name
	*Iris lactea var. chinensis* (Fisch.) Koidz.	–	Named as place name
**Belief**	*Juniperus rigida* Sieb. et Zucc.	Branchlet	To substitute for incense in religious activity or sacrifice

### Mongolian names of plants in MAKK

In MAKK, Kozlov transliterated the Mongolian name of plants in Russian. However, because of the changes of times, dialect accents and transliteration deviations, the pronunciations for the Mongolian names of plants recorded by Kozlov are moderately different from the standard pronunciations and dialects in current Mongolia. Hence, we have carried on identification, analysis and generalization of these names. We learn that there are two cases about the Mongolian folk names of plants enrolled in MAKK as follows: (a) The Mongolian name of a plant is explicitly identified, that is, Mongolians call a plant by its name. In the meantime, the Mongolian names of plants are written as two types. The first refers to the scientific name of the plant, and the second does not showcase its scientific name, only described by its own habitat and characteristic. (b) The Mongolian name of a plant is not clearly pointed out, while spelled in Russian according to Mongolian pronunciation, but its corresponding scientific name is given. There are two categories of recognized vocabulary: the first is not Russian but Mongolian glossary. The second is that the vocabulary can be attributed to both Mongolian and Russian words, which have the same meanings.

A great deal of achievements have been made on the aspect of the naming of plants in the Mongolian folk research, such as Shan Chen [66], Khasbagan [1, 7, 67–70], Soyolt [36], Wuren Hu [62, 63], Yanying Zhang [71, 72], and Urtnasan Mandakh [73]. On this basis, the Mongolian names of plants in MAKK are analyzed as follows.

### Verification of Mongolian folk names of plants

In texts of two versions, it was recorded that Mongolian names of *Populus euphratica* Oliv. are “Тограк”, while its Mongolian name was set down as “Тограк или Хайлис” in the appendix of MAKK in 1923. In order to clarify the fact, we paid a deep visit to the place (Alxa) where Kozlov recorded *P. euphratica* Oliv. All the local Mongolians had a distinct recognition due to the entirely different features of two plants. In the light of interviews, Alxa Mongolians have been calling *P. euphratica* Oliv. as “/tɔ:rɔi/” and *Ulmus pumila* L. as “/xɑils/”.

The Mongols lived in diverse regions have some differences towards the same plant. The Mongolian folk name of *Amygdalus mongolica* (Maxim.) Ricker is called as “Хату-хара” in MAKK. Communicating with Mongolian herdsmen in Alxa, we got a thorough knowledge of four Mongolian terminologies on *A. mongolica* (Maxim.) Ricker: “/xɑtʊ: xɑr/”, “/ʊlɑ:n bʊɪls/”, “/xɑr mɔd/”, and “/xɑr bʊt/”. The first is diffusely employed in the entire Alxa Region, the second in Ordos area, the third merely in the vicinity of Alxa Jartai, and the last in most districts of the Alxa Left Banner. Looking up reference books, we discover that the Mongolian name of *A. mongolica* (Maxim.) Ricker recorded in *Flora of China* [46] and *Flora of Inner Mongolia* [74, 75] is described as “”. By the contrast, “” is the formal name and “” is the vulgar name in the *Mongolian-Chinese-Latin Names of spermatophyte* [76]. The results demonstrate the majority of Mongolians existed in Alxa call *A. mongolica* (Maxim.) Ricker as “”, on the contrary, “” is expressed as only one folk name of *A. mongolica* (Maxim.) Ricker in the Ordos districts. All the proofs declare the formal name of *A. mongolica* (Maxim.) Ricker is derived from Ordos areas. Meanwhile, the current usage situations of Mongolian names represent the traditional Mongolian names of plants came from diverse regions have been preserved and inherited comparatively integrated.

There are a crowd of Mongolian folk names for the identical plant in the same area. For example, the Mongolian folk names of *Nitraria tangutorum* Bobr. are depicted as “/xɑrmɑg/”, “” /bo:rog/ and “/ʊsn usəg/”, moreover, *Iris lactea var. Chinensis* (Fisch.) Koidz. can be named as “/tʃæxʲɑrmɑ/” and “/tʃæxʲɑrmɑ/”. Kozlov merely kept a record of one of them in MAKK. In terms of the phenomenon, the subjective reason is that Kozlov did not pay excessive attention to the Mongolian names of plants. The objective aspects are as follows: one is that the range of Kozlov’s investigation was small and limited. The other is that perhaps individual Mongolian names did not exist hundreds years ago. With the cultural exchange and social evolution, Mongolian folk names of plants have been continually enriched. Nevertheless, it is sufficiently proved that the Mongolian names of plants recorded in MAKK are traditional folk names full of some historical and cultural value.

All in all, Mongolia culture is filled with multifarious traits of various regions and tribes in terms of plant naming. The Mongolian names of plants (Table 1) are constantly used in local area by interviews. The phenomenon indicates the Mongolian folk names of plants served as a conventional culture are almost perfectly preserved and inherited in the research districts.

Three plants additionally recorded in MAKK have the same Russian names as the Mongolian ones, which are *Spiraea mongolica* Maxim.*, Caragana* Fabr. and *Rubia cordifolia* L., respectively. In contrast, their Russian and Mongolian names are “Таволга” and “[tabilgan_a]”, “Карагана” and “[xargan_a]”, as well as “Марена” and “[marin_a]”, individually. “” and “” are not traditional folk names of the Mongolian nationality because they have not been visited or relevant recorded. Since the Russian and Mongolian names exist in a borrowing relationship of these two plants, which are not the study contents of the paper, they are only pointed out but not included in Table 1.

### The correspondence between the Mongolian folk and scientific names of plants

*Amygdalus mongolica* (Maxim.) Ricker*,* a scientific name, corresponds with four Mongolian names: “”, “”, “” and “”. Meanwhile, *Nitraria tangutorum* Bobr. possesses three Mongolian names, *and Iris lactea* var. *Chinensis* (Fisch.) Koidz. owns two Mongolian names (Table 1). There is a “multitude-to-one” relationship between Mongolian folk name and scientific name of a plant. In view of this, it proves that the phenomenon of synonym consists in the Mongolian folk naming of plants, that is, there are two or more Mongolian names for the identical plant.

It is a “one-to-one” relationship between Mongolian and scientific names of *Populus euphratica* Oliv., *Ulmus pumila* L., *Agriophyllum squarrosum* (L.) Moq., *Achnatherum splenden* (Trin.) Nevski, together with *Kobresia tibetica* Maxim. (Table 1). Noteworthily, the Mongolian folk names of all five plants are the primary name (PN) and have no other meanings. It demonstrates the Mongolian folk classification of certain plants have attained the level of species over a hundred years ago.

However, “Карагана ()” is the Mongolian folk name of *Caragana* Fabr. in MAKK. “” corresponds to many plants of the genus *Caragana* Fabr.. Moreover, *Kobresia tibetica* Maxim. is named as “Мото-ширик (/mɔd ʃirəg/)” in MAKK. In modern plant taxonomy, “” is exclusively referred to *Carex* L. By contrast, both *Carex* L. and *Kobresia* Willd. are thought as “” without strict distinction among Mongolian folk. It follows that there is a “one-to-multitude” correspondence between Mongolian folk names and scientific names. First of all, it is verified that the folk naming of plant lies in the phenomenon of homonym, that is, the same Mongolian name refers to two or more plants. Furthermore, the folk classification level of some plants, being at the standard of genus or family, is considerably far from the current levels.

Analyzing the relationship between Mongolian folk names and scientific names, Mongols have a profound comprehension of some plants and a relatively high level of sorting. Nevertheless, it is slightly shallow and inferior for the understanding of other plants and the level of classification. The reason for the condition has something to do with the usage value of plant resources and the closeness of certain plant in the daily life of Mongolians.

### Structures and types of Mongolian folk names of plants

Structurally, Mongolian folk names of plants in Table 1 can be classified as simple primary name, complex primary name and secondary name [77, 78]. Among them, “”, “”, “/sʊlhir/”, “”, “”, “”, “/dərs/”, “”, and “” belong to simple primary name, specifically referring to the relevant plants, and having no other meaning. “”, “”, and “” are complex primary name, consisted of “common word + common word”. “”, “” and “” are secondary name and made up of “modifier + simple primary name”, among which “” and “” are modifier, in the meantime, “”, “”, and “” are simple primary name. This is very similar to Linnaean binomial nomenclature, where modifier is equivalent to specific epithet, but existing in a different position. It declares the concepts of genus and species below genus [67].

In the plant names of Mongol folk, primary names have the most cultural significance. However, secondary names indicated the existence of folk generic and have important meanings for folk classification. The means and cases of Mongolian folk naming of plants have exhibited a vigorous influence and a reference value on drafting Mongolian names in contemporary botany.

### Meaning and naming basis of Mongolian folk names of plants

It is one of the vital evidences for naming plants based on the morphological features and properties.

The Mongolian name of *Amygdalus mongolica* (Maxim.) Ricker is “” in MAKK. In the Mongolian language, “” means hard, and “” refers to black. Resulting from hard wood of branches and gray black bark, along with looking black of thickets from a distance, Mongolian people name *A. mongolica* (Maxim.) Ricker with the vocabulary describing its morphological traits in the folk.

Taking *Nitraria tangutorum* Bobr., as another example, its Mongolian name is “”. The meaning of “” is water-rich, and “” means sour. Such plant is so named because the fruit of the plant is a berry, being filled with juicy and having a sour taste. Similarly, “”, “”, “”, and “” are called after in the same way.

On the basis of naming, it is quite common to nominate plants in line with their morphological features and natures in Mongolian folk [69, 79]. Analyzing the structure, implication, and basis of plant naming, for naming and classifying of plants, the Mongolians should have indigenous rules and systems [66, 67]. It is formed and inherited for the special knowledge during the Mongolians’ long-term acquaintance and practice of traditional utilization of plants.

### The relationship between language culture and plant names of the Mongols

The plant names of Mongolian folk in Alxa, such as “,,”, mentioned in MAKK do not exist in the eastern region of the Mongolian Plateau. The authors think that it is a special vocabulary, for denominating the plant is created by the Mongolians lived in Alxa under the floristic conditions of this region. In this sense, desert plants enrich the Mongolian language, which produce a significant influence on Mongolian culture.

### Usage information of plants in MAKK

The Mongolians were nomadic people and not engaged in agricultural production at that time, whose conventional experience of plant usage was about wild plants. The utilization of cultivated plants by the Mongolian aristocrats in Dingyuanying was not within the scope of the article. The classical usage of plants enrolled in MAKK is classified by Mongolian folk according to their purposes, mainly involving edible, forage, medical, fuelwood, building, cultural, and other aspects’ usage (Table 2).

It can be seen that a plant may possess a variety of purposes; meanwhile, different plants of the same use have different parts and ways of utilization (Table 2). Based on the purposes of plants, do the following analysis.

### Edible

**Fig. 6:**
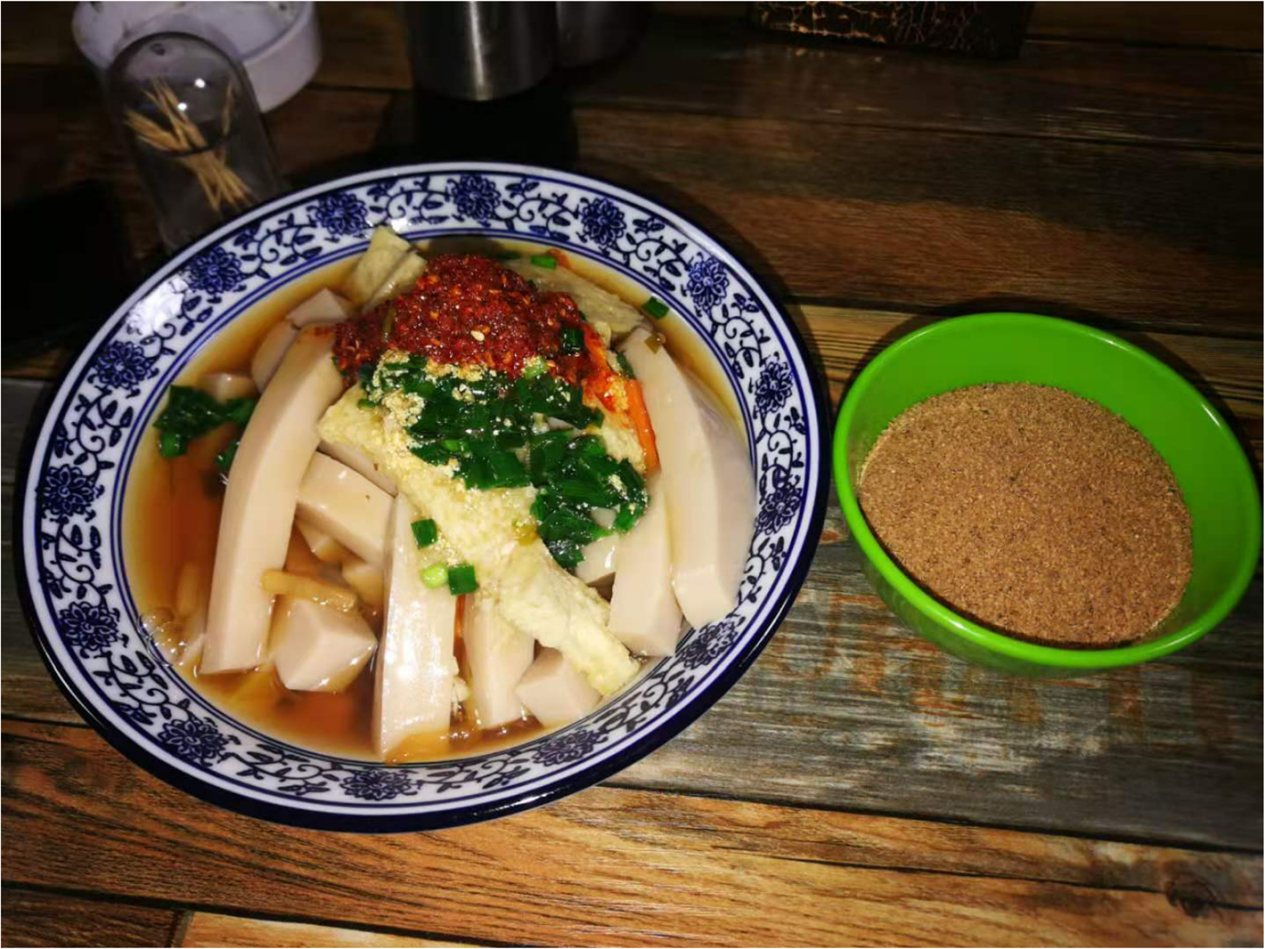
Seeds of *Agriophyllum squarrosum* (L.) Moq. and the jelly made from them. (Taken by Su Yun in Alxa)

**Fig. 7:**
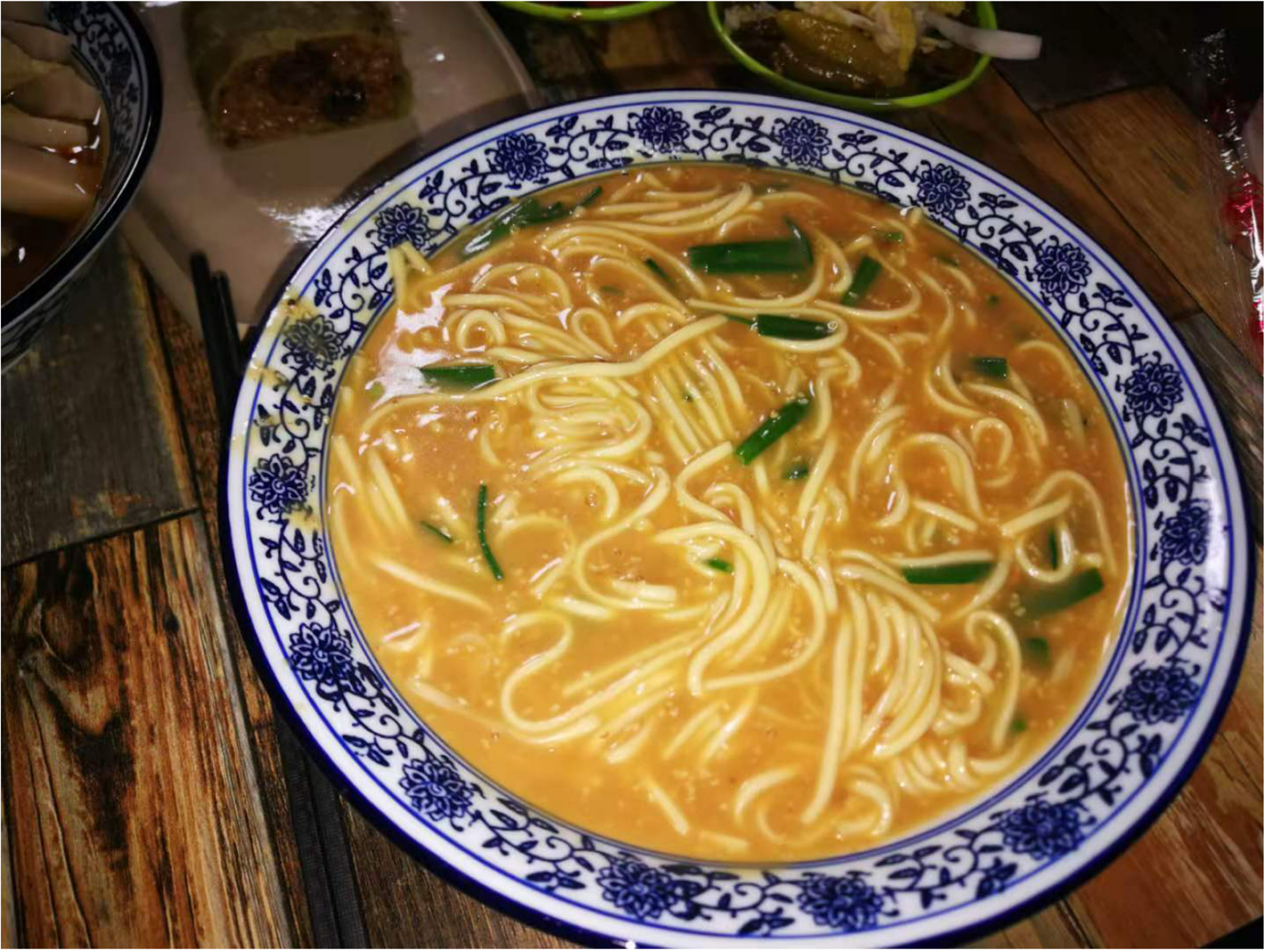
Tiaohuo made of noodles and seeds of *Agriophyllum squarrosum* (L.) Moq. (Taken by Su Yun in Alxa)

*Agriophyllum squarrosum* (L.) Moq. which prefers to grow on the leeward slopes of dunes is an annual herb of chenopodiaceae. It is a kind of familiar psammophyte in desert areas of northern China. The seeds of *A. squarrosum* (L.) Moq. incorporated in MAKK are the usual wild grain plants of Mongolian people in Alxa, Gansu, and other desert areas. In autumn, the local herdsmen harvest its seeds, fry them, grind them into powder, and make them into Zanba (roasted barley flour) with butter and dairy products, or stir fry them with butter and boil it together with brick tea to make a kind of rice tea. The eating methods above are the chiefly edible way of Mongolian people in Mongolia [80]. The seeds of *A. squarrosum* (L.) Moq.*,* known to Han nationality as Shami, have been conventionally acted as grain substitutes [81, 82]. Nowadays, people in these regions remain the habit of eating Shami. The ways and practices of eating Shami have increasingly become multiform with the evolution of society [59]. Making its powder into jelly, we can taste better after seasoning (Fig. 6). A dish of mutton soup is cooked by adding Shami and noodles, which is called Tiaohuo (Fig. 7).

**Fig. 8:**
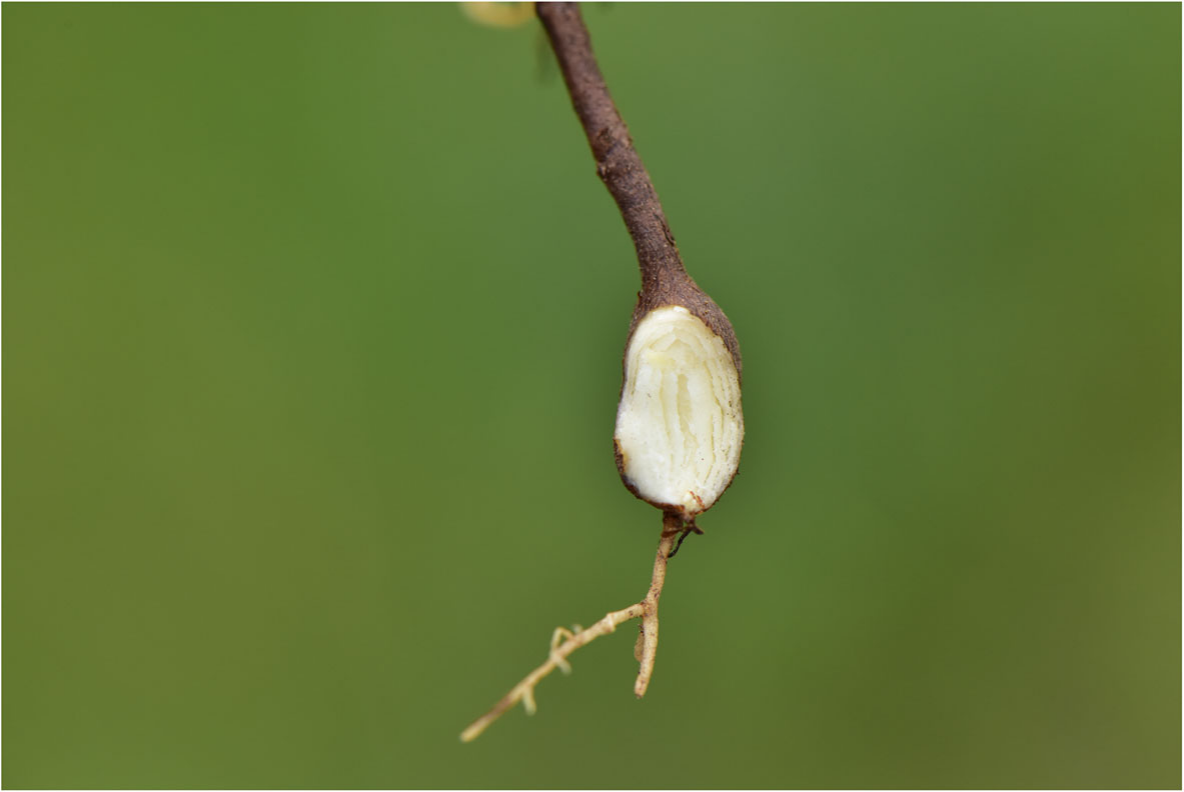
The root of *Potentilla anserina* L. in Qinghai. (Taken by Zhu Xinxin in Menyuan, Qinghai Province)

**Fig. 9:**
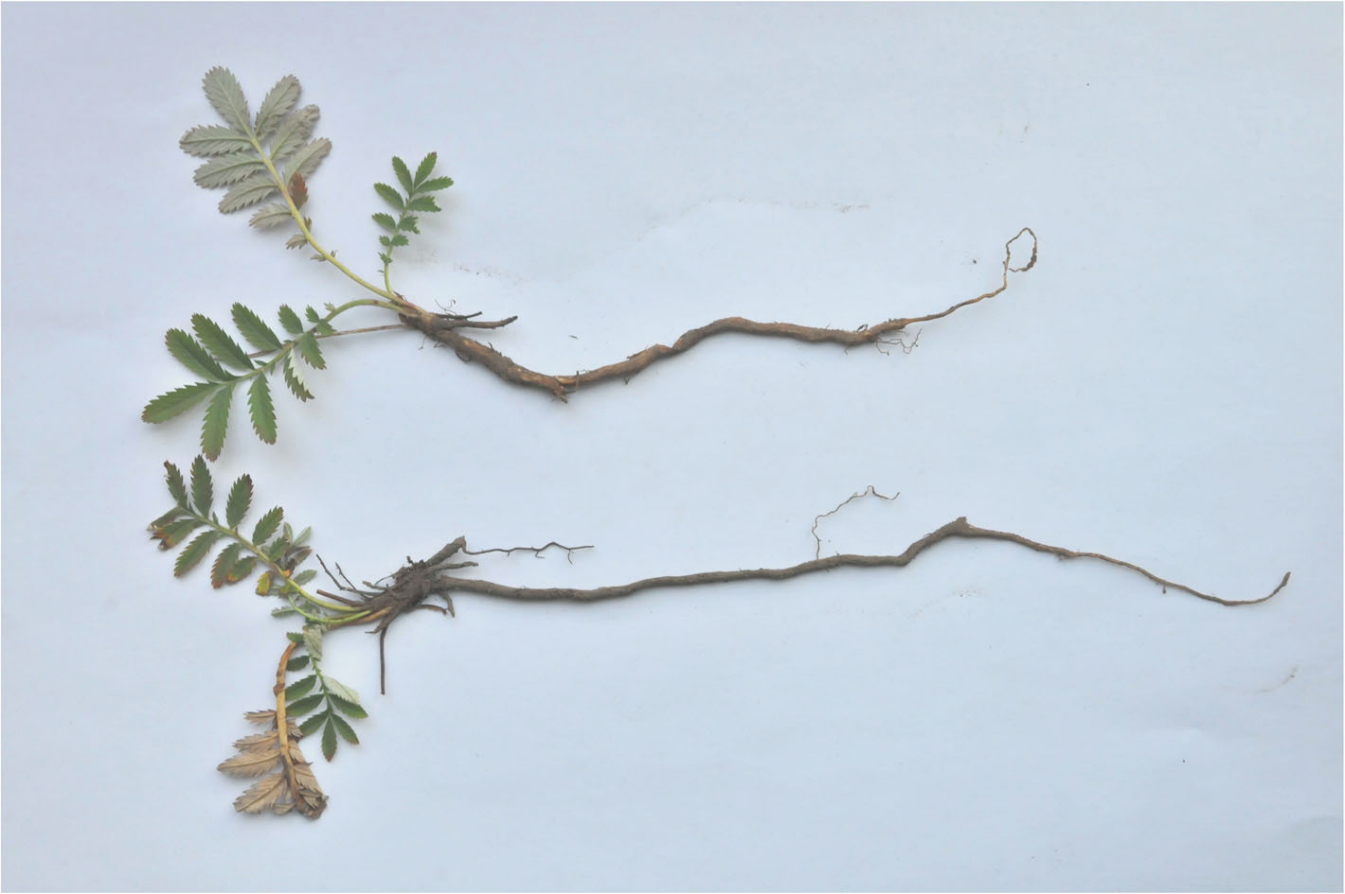
*Potentilla anserina L.* in Inner Mongolia. (Taken by Liu in Hohhot, Inner Mongolia)

*Potentilla anserina* L. is a perennial herb of Rosaceae and their roots expand and grow into spindle-shaped or oval tuberous roots in the alpine regions of Gansu, Qinghai and Xizang (Fig.8). The tubers are rich in starch and used for edible and medicinal [46, 74]. It was recorded in MAKK that the root tubers of *P. anserina* L. are excellent cuisines and are noted for its delicacy throughout the Qinghai-Tibet Plateau [17, 18]. However, the root of *P. anserina* L. is not swollen or tuberous (Fig. 9), so Mongolians living in the Mongolian plateau are not able to regard it as food. In addition, there is no relevant literature on its consumption.

By means of interviews, residents of Qinghai and other places, including Mongolians and other ethnic groups, often eat *P. anserina* L. in diversiform ways such as being steamed or boiled, and speak highly of it nowadays. Unfortunately, there was no record about its edible ways at the time in MAKK.

### Graziery

Since animal husbandry is the primary industry of the Mongols, the use of plants is primarily for forage*. Haloxylon ammodendron* (C. A. Mey.) Bunge belongs to the small chenopodiaceae arbor, mostly spreading on the dune, saline-alkali desert, river sandy land, and so on [83], and it is one of the indispensable forage plants for local people. It is also noted in MAKK that camels like eating the young branches and leaves of *H. ammodendron* (C. A. Mey.) Bunge [17, 18]. The facts showcase local people have long been concerned about the palatability of forage plants for livestock. The investigation result of Wuren Hu [65] attests the plant is a local high-quality plant for feeding camels.

**Fig. 10:**
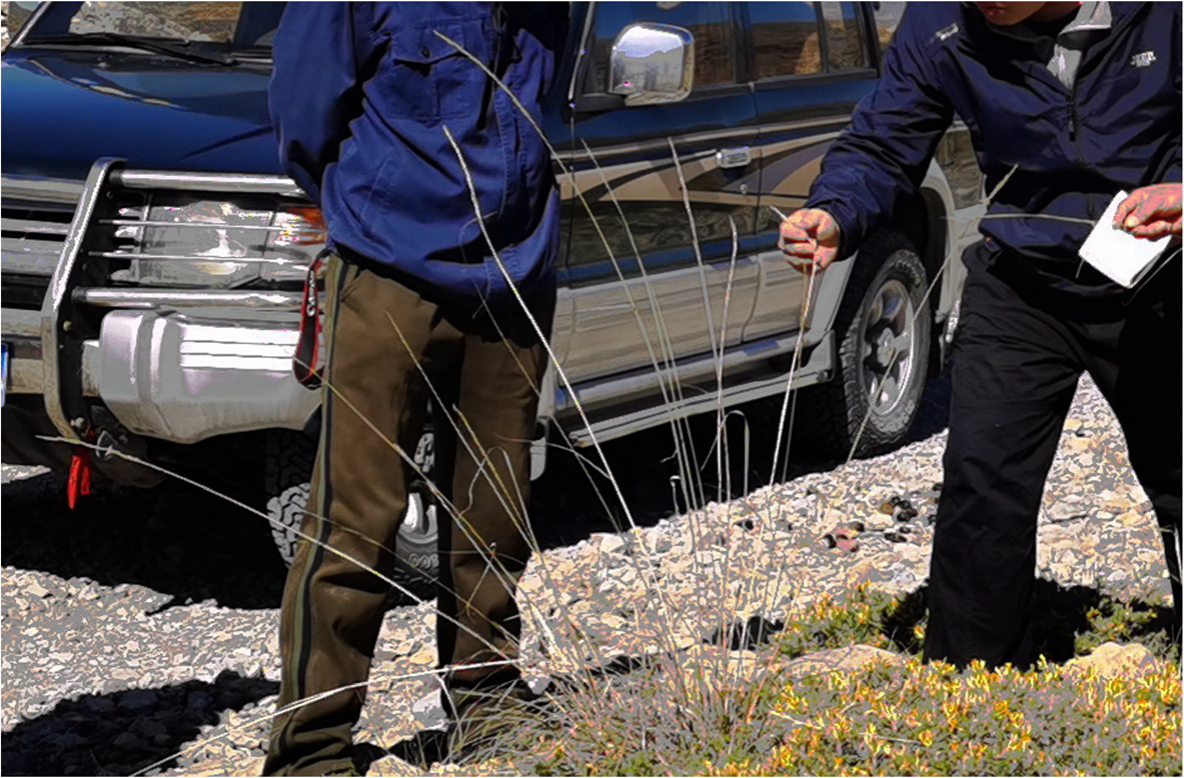
*Achnatherum inebrians* (Hance) Keng. (Taken by Wuriheng at Xiaodiangou of Ho-lan Mountains)

Excluding forage, there are other records of precautionary knowledge about poisonous or harmful plants written in MAKK. As for the note of *Achnatherum inebrians* (Hance) Keng, “Agvan, a Mongolian guide, recommended us several poisonous *A. inebrians* that can cause horses to fall ill or even die after ingestion.” [17, 18] (Fig. 10) It manifests that the Mongolians have accumulated experience of guarding against this plant in the course of grazing.

### Fuelwood

*Haloxylon ammodendron* (C. A. Mey.) Bunge is an essential fuel plant for Mongolians living in the desert and Gobi areas. In accordance with MAKK, dried cow dung contains fibers originated from many plants and is the prime fuel of the Mongolians. The Torgut Mongols in Alxa have another significant energy plant-*H. ammodendron* (C. A. Mey.) Bunge. It usually forms a wide range of open forest in desert regions, which plays a crucial role for fixing dune, and its wood is a very fine choice for fueling [17, 18]. It has been utilizing in daily lives of Mongolian herdsmen at present.

### Building material

**Fig. 11:**
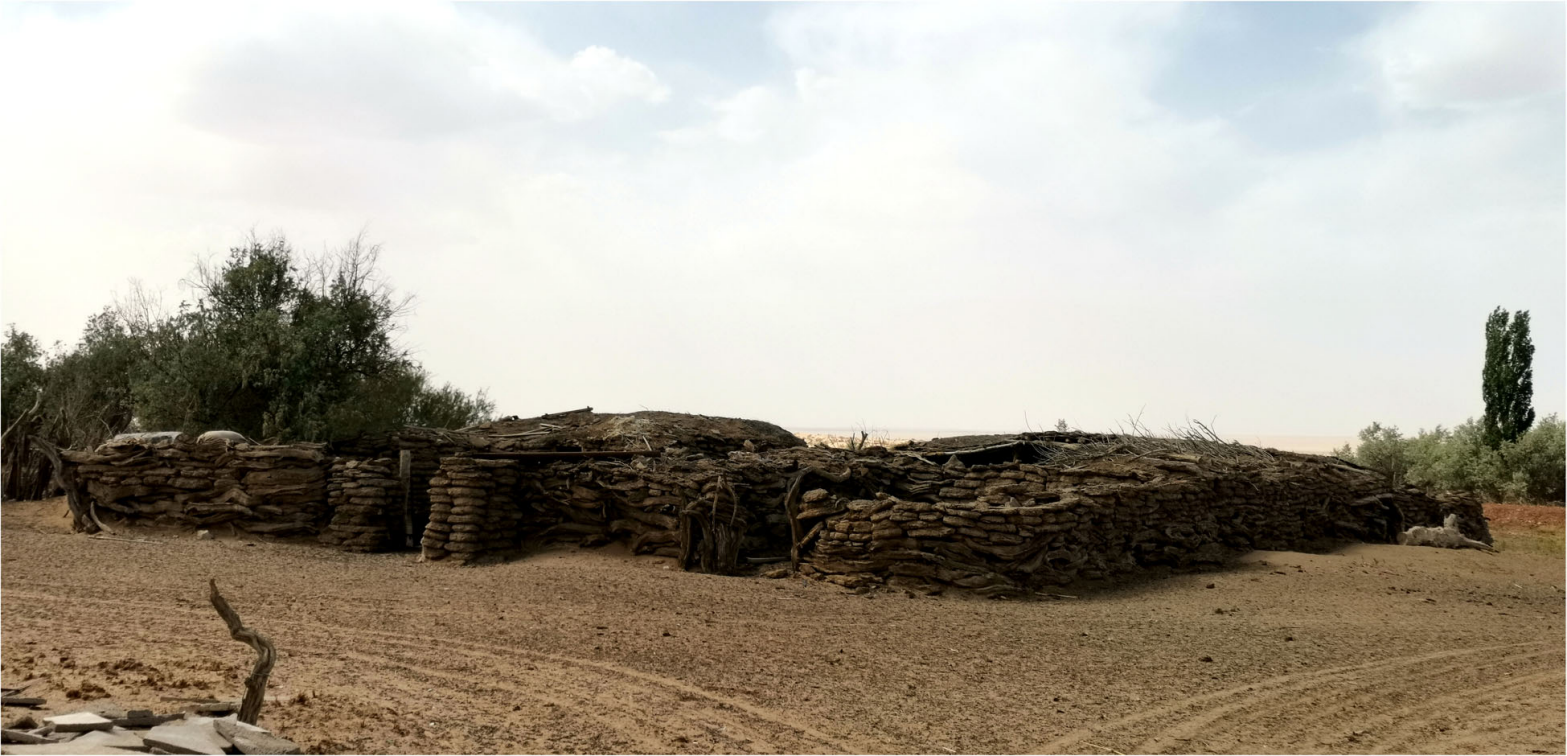
Stock barns made of *Haloxylon ammodendron* (C. A. Mey.) Bunge. (Taken by Liu in Ejina)

**Fig. 12:**
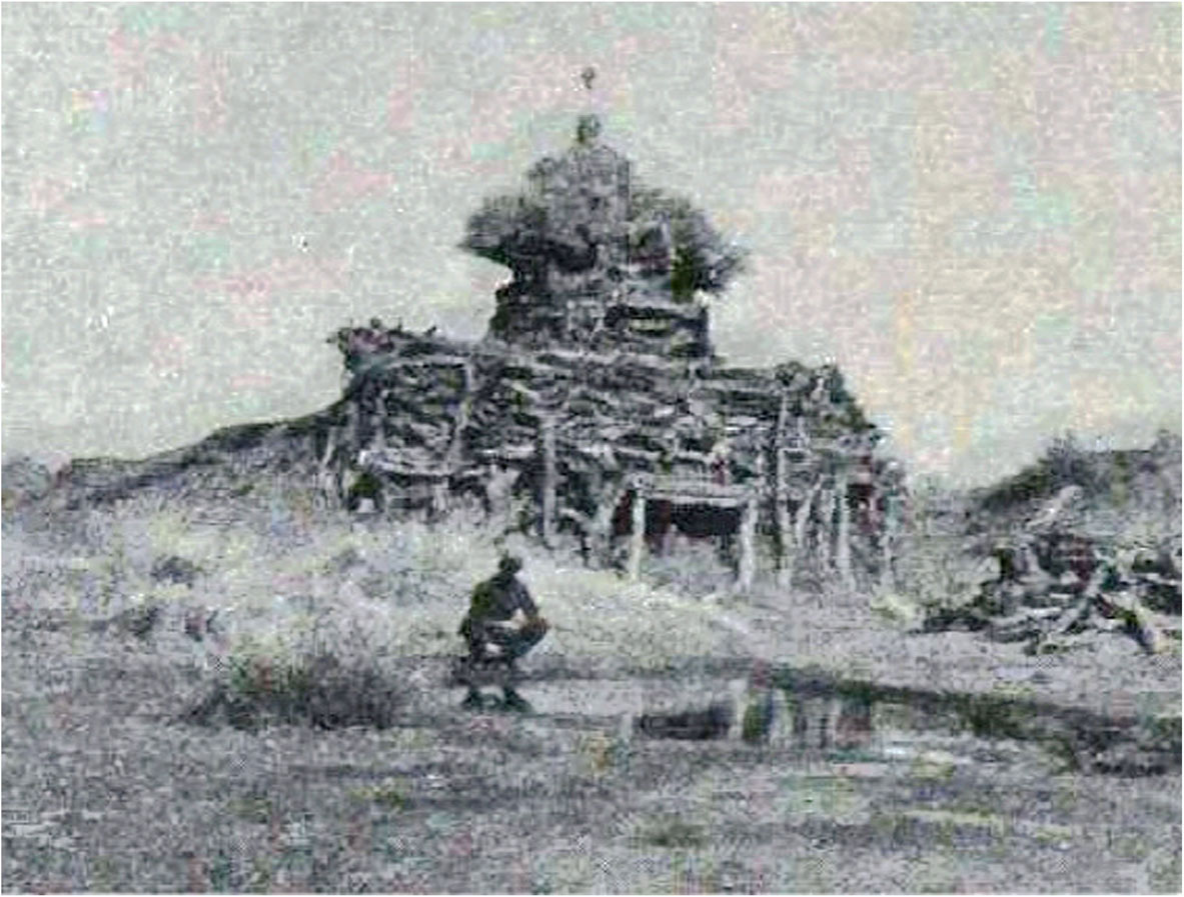
Obo made of *Haloxylon ammodendron* (C. A. Mey.) Bunge. (Copied from the 1923 edition MAKK)

**Fig. 13:**
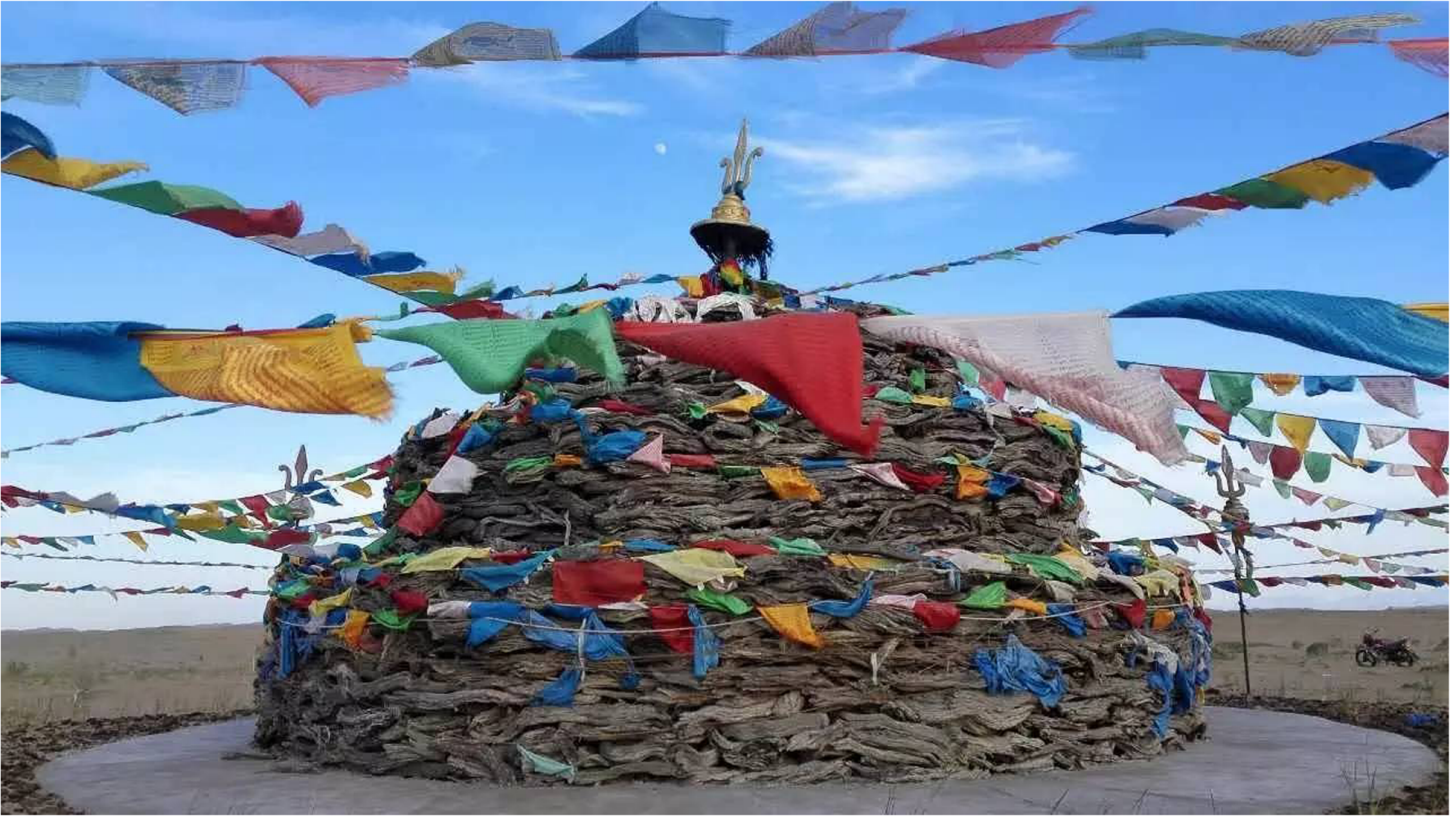
Kente-Obo made of *Haloxylon ammodendron* (C. A. Mey.) Bunge (http://img.mp.itc.cn/upload/20160623/6a6fc47493e94cf38c6446bffce5f8f3_th.jpg)

*Haloxylon ammodendron* (C. A. Mey.) Bunge and *Phragmites australis* (Cav.) Trin. ex Steud are the main plants in architecture mentioned in MAKK. *Haloxylon ammodendron* (C. A. Mey.) Bunge is a small tree, whose wood is strong and brittle, and its trunk and branches are often used to build walls or stock barns (Fig. 11). There is a convention of sacrificing Obo in Mongolian folk. The common Obo is made of piled stones. In addition, there is an Obo consisted of stacked branches of plants mentioned in MAKK (Fig. 12). These plant Obos are mainly made from the branches of trees such as *H. ammodendron* (C. A. Mey.) Bunge, *Populus euphratica* (Cav.) Trin. ex Steud *and Ulmus pumila* L.*.* It is reported that this phenomenon is still comparatively common in Alxa today (Fig. 13).

**Fig. 14:**
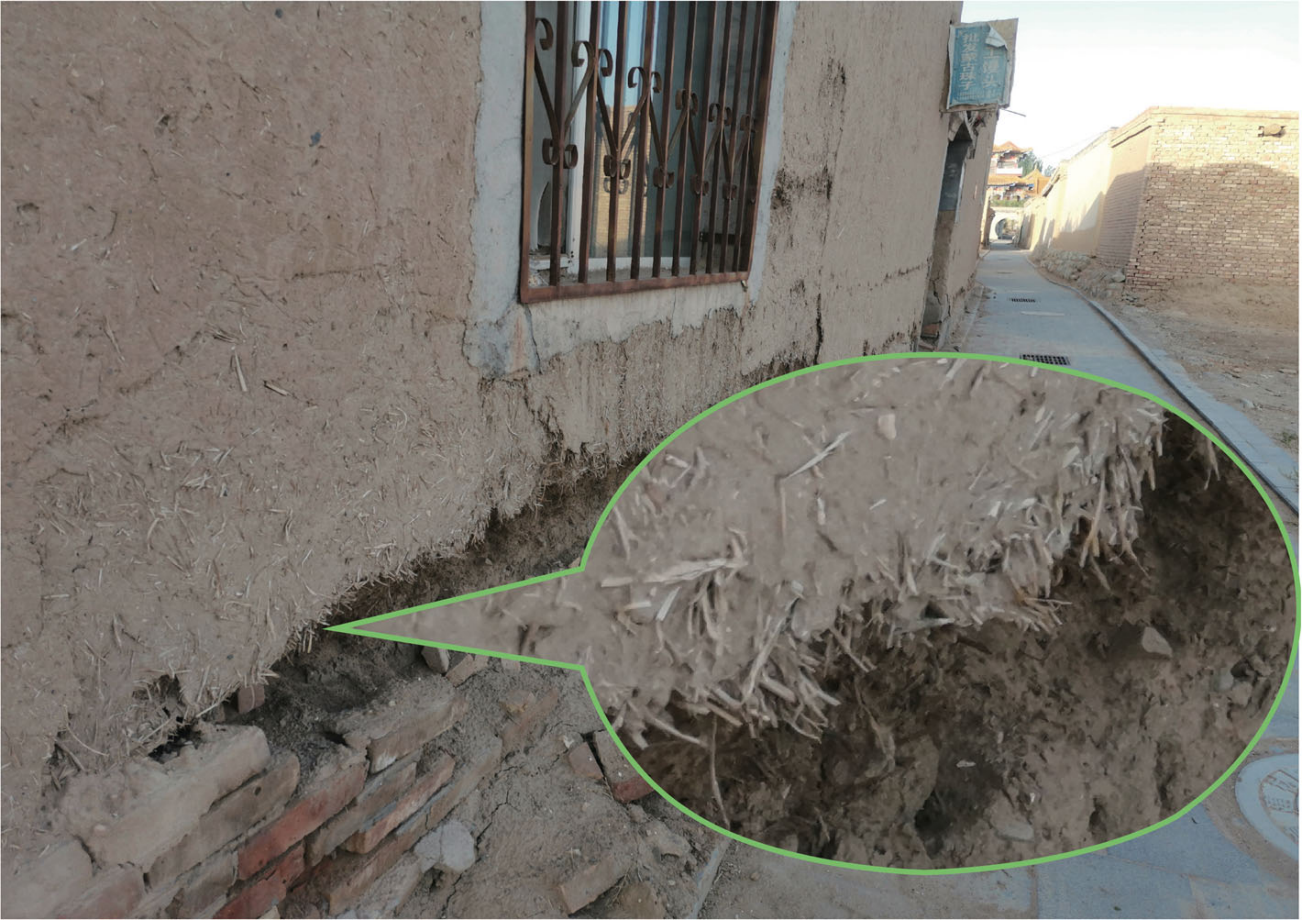
A dobe wall mixed with straws of *Phragmites australis* (Cav.) Trin. ex Steud. (Taken by Liu in Alxa Left Banner)

As a perennial herb, the haulm and leaves of *Phragmites australis* (Cav.) Trin. ex Steud are rich in fibers, which can be mashed and mudded, and then extensively used for constructing temples. The application of building residential houses is probably affected by the farming culture of the Han nationality in the process of cultural exchange, rather than the originally traditional method created by the Mongolians (Fig. 14).

### Toponym

**Fig. 15:**
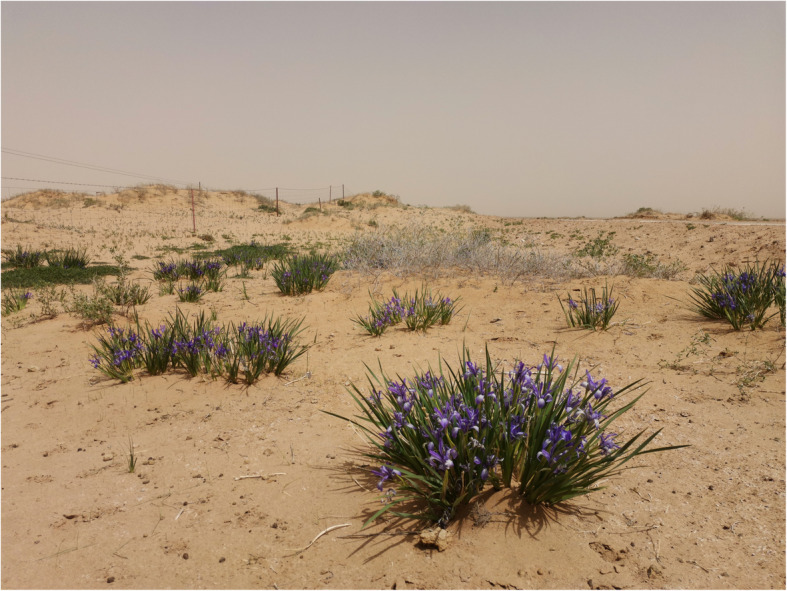
*Iris lactea* var. *chinensis* (Fisch.) Koidz. (Taken by Liu at Zabusar nearby Цакэлдэктэ-худук)

There is an intimate relationship between plants and human geography of Mongolian folk, and the most prominent character is that the naming of many toponym is directly related to the distribution of plants [73]. For example, Kozlov’s expedition was stationed in a place called “Дурбун-мото кородезь ()”, which means “the well with the four trees.” It is mentioned in MAKK that the locals name it according to the actual situation that the well is surrounded by four flourishing old *Ulmus pumila* L.. Likewise, the Mongolian name of *Iris lactea* var. c*hinensis* (Fisch.) Koidz. is “”. The zone of the specimens collected is called as “Цакэлдэктэ-худук ()”, referring to “the well with Malan flowers”. There is a sea of blue *I. lactea* var. *chinensis* (Fisch.) Koidz. grown in full bloom around the well, leading to the locals name it after “/tʃæxʲɑldɑgtɑi xʊdʊg/”. The author conducted a survey of the record site in MAKK and found that there existed a wide range of *I. lactea* var. *Chinensis* (Fisch.) Koidz. nearby (Fig. 15).

It can be seen that the distribution of plants is a vital factor in the naming of toponym, which carries a great deal of information about the historical distribution of plants. Through the interpretation of place names, we can infer the historical situation of a certain plant distribution, which can provide historical clues for the study of local natural ecological conditions.

### Belief

Among religious beliefs and sacrificial activities, Mongolian folk’s utilization of plants can be found everywhere. Some of musical instruments are made up from birch (*Betula* sp.) in Tibetan Buddhism. Additionally, it is also noted in MAKK that the monks would light a small bundle of juniper branches instead of burning incense during the requisite ceremonies.

Investigations show that some ways of using plants mentioned above are no longer common in the Mongolian folk, or even have disappeared. We can acquire the enlightenment that not only the current folk knowledge on plants should be recorded and preserved, but also the traditional knowledge enrolled in the literature should also be explored and studied, further passed on as a kind of culture.

## Conclusion

The records of local botanical knowledge of the Mongols are veritable and reliable in MAKK through textual research. We can draw the following conclusions from the above statement.

- Through the analysis of the structure, meaning and name basis of Mongolian plant names, it is found that apart from the inheritance of proper names, Mongolian folk may well possess a particular set of naming rules and classification system for plants, which needs further systematic research.
- In terms of plant nomenclature, the Mongolian name of the same plant in different regions is different, and the same plants in different tribes in the same area are also different. Plants are mainly used in Mongolian daily life and other aspects such as architecture, entertainment, belief and prevention. It demonstrates the multiformity of traditionally Mongolian botanical knowledge.
- Local botanical knowledge is changing with the evolution of the folk culture. Meanwhile, due to the enrichment or loss of local botanical knowledge, local folk culture also undergoes a transformation. These manifest that the local botanical knowledge and folk culture of the Mongols have mutual influence and intimate interdependence.
- With the development of society, the change of the lifestyle together with the interference of diverse human factors, the local knowledge on plants is disappearing at an alarming speed. Hence, it is extremely vital and urgent to sort out, protect, and inherit the relevant information enrolled in the literature. In view of this, we suggest that it should be reasonably protected by various forms, for example, recording, reporting and re-research.
- In the long-time interactions with plants, Mongols have gathered a large amount of special botanical knowledge and utilization experience, having a formation of cultural tradition with local traits. The Mongolian local knowledge on 14 plants mentioned in MAKK confirms the traditionality of the local knowledge and enriches the content of Mongolian ethnobotany. According to the survey, a large amount of naming and utilization methods recorded by Kozlov are still in use. They offer faithful information and momentous historical data for the study of Mongolian ethnobotany. What is more, the plant records more than 100 years ago are of certain reference value for the research of ecology, flora and botanical history.

## Data Availability

All data generated or analyzed during this study are included in this published article and its supplementary information files.

## Abbreviation

MAKK: Mongolia and Amdo and the Dead City of Khara-Khoto
